# Combined Elevation of *microRNA-196a* and *microRNA-196b* in Sera Predicts Unfavorable Prognosis in Patients with Osteosarcomas

**DOI:** 10.3390/ijms15046544

**Published:** 2014-04-17

**Authors:** Chun Zhang, Cong Yao, Haopeng Li, Guoyu Wang, Xijng He

**Affiliations:** 1 Department of Orthopedic Surgery, the Second Hospital Affiliated to Xi’an Jiao Tong University School of Medicine, Xi’an 710004, Shaanxi, China; E-Mails: zyt1117@126.com (C.Z.); lhp-3993@163.com (H.L.); wgy1509@126.com (G.W.); 2 Department of Aesthetic Plastic Surgery, the Second Hospital Affiliated to Xi’an Jiao Tong University School of Medicine, Xi’an 710004, Shaanxi, China; E-Mail: 1113zyt@163.com

**Keywords:** osteosarcoma, *microRNA-196a*, *microRNA-196b*, serum, clinicopathological features, overall survival, disease-free survival

## Abstract

**Aim:**

To investigate whether the aberrant expression of *microRNA (miR)-196a* and *miR-196b* can be used as potential prognostic markers of human osteosarcoma.

**Methods:**

Quantitative real-time reverse transcriptase-polymerase chain reaction (qRT-PCR) analysis was performed to detect the expression levels of *miR-196a* and *miR-196b* in osteosarcoma tissues and patients’ sera.

**Results:**

Expression levels of *miR-196a* and *miR-196b* in osteosarcoma tissues were both significantly higher than those in noncancerous bone tissues (both *p* < 0.001), in line with which, the serum levels of the two miRNAs were also markedly upregulated in patients with osteosarcomas compared with healthy controls (both *p* < 0.001). Then, the elevation of serum *miR-196a* and *miR-196b* levels both more frequently occurred in osteosarcoma patients with high tumor grade (*p* = 0.008 and 0.01, respectively), positive metastasis (*p* = 0.001 and 0.006, respectively) and recurrence (*p* = 0.001 and 0.006, respectively). Moreover, high serum *miR-196a*, high serum *miR-196b* and conjoined expression of *miR-196a*/*miR-196b* were all independent prognostic factors for OS (overall survival) and DFS (disease-free survival) of osteosarcoma patients.

**Conclusion:**

Our present data indicate the involvement of *miR-196a* and *miR-196b* upregulation in the pathogenesis of osteosarcoma. More importantly, the altered levels of circulating *miR-196a* and *miR-196b* might have great potential to serve as novel and non-invasive prognostic factors for this malignancy.

## 1. Introduction

Human osteosarcoma represents one of the most common primary sarcomas often originating in the metaphyses of long bones [1]. It has an incidence of four to five cases per million worldwide and is a leading cause of cancer-related death in children and young adults [2]. Due to the high rate of systemic spread, cure is rare after surgical treatment alone. With the introduction of combinatorial chemotherapy, the five-year survival rate of patients with osteosarcoma has dramatically improved over the past decades to approximately 60%–70% [3]. However, there still are a significant proportion of osteosarcomas patients with poor response to chemotherapy, and they have a high risk of local relapse or distant metastasis even after curative resection of the primary tumor and intensive chemotherapy [4]. In addition, the molecular events which initiate and propagate osteosarcomagenesis remain obscure. Thus, the identification of novel and efficient molecular markers is extremely necessary for the improvement in treatment of osteosarcoma and for the prediction of clinical outcome of patients with this malignancy.

MicroRNAs (miRNAs) are a class of small, endogenous, non-coding RNA molecules with highly conserved sequences across species in plants, animals, and DNA viruses [5]. These molecules function as posttranscriptional regulators by inhibiting gene expression through either cleavage of the target mRNAs or translational repression [6]. It has been estimated that miRNAs can regulate as much as 60% of the human protein coding genes [7]. The active miRNA is preferentially incorporated into the RNA-induced silencing complex, which recognizes specific mRNA targets through complementary binding, mainly mediated through the “seed” sequence. miRNAs with identical seed sequences may target the same mRNA, and are grouped into miRNA families. On the other hand, the same miRNA may target multiple mRNAs [8]. Functionally, miRNAs have been demonstrated to be integral components of many normal biological processes involving cell proliferation, differentiation, apoptosis, and stress resistance [9]. More importantly, accumulating studies also show that miRNAs can be dysregulated in various pathological processes, such as human cancers [10]. They function either as oncogenes or as tumor suppressors depending on the role of their target mRNAs [11]. An increasing number of research have demonstrated that miRNA expression profiles can distinguish tumors from corresponding normal tissues, as well as by their developmental origin and differentiation state [12]. Thus, we hypothesized that the selected miRNA markers confirmed in clinical samples compared to bone may provide new insight into the complex genetic mechanisms of osteosarcoma development and progression.

Human miR-196 cluster consists three different members including *miR-196a-1*, *miR-196a-2*, and *miR-196b*, and appears to be expressed from intergenic regions in HOX (homeobox) gene clusters, which have been recognized as major transcription factors involved in embryogenesis, organogenesis and oncogenesis [13]. The *miR-196a-1* gene is located on chromosome 17 (17q21.32) at a site between *HOXB9* and *HOXB10* genes, the *miR-196a-2* gene is located at a region between *HOXC10* and *HOXC9* on chromosome 12 (12q13.13), and the *miR-196b* gene is located in a highly evolutionarily conserved region between *HOXA9* and *HOXA10* genes, on chromosome 7 (7p15.2) in human beings and chromosome 6 (6qB3) in mice [14]. *miR-196a-1* and *miR-196a-2* genes transcribe the same functional mature miRNA sequence, whereas *miR-196b* gene produces a small RNA, which differs from the sequence of miR-196a by one nucleotide [14]. Both *miR-196a* and *miR-196b* have been demonstrated to play a crucial role in normal cell differentiation, proliferation, and in tumorgenesis of various cancer types [15]. Especially, Namløs *et al*. [16] observed the upregulation of the two miRNAs in osteosarcoma cell lines relative to normal bone. However, their clinical significance remains unclear. Thus, the aim of this study was to investigate whether the aberrant expression of the two miRNAs can be used as potential prognostic markers of human osteosarcoma.

## 2. Results and Discussion

### 2.1. Upregulation of miR (microRNA)-196a and miR-196b in Human Osteosarcoma Tissues and Patients’ Sera

**Figure 1.**
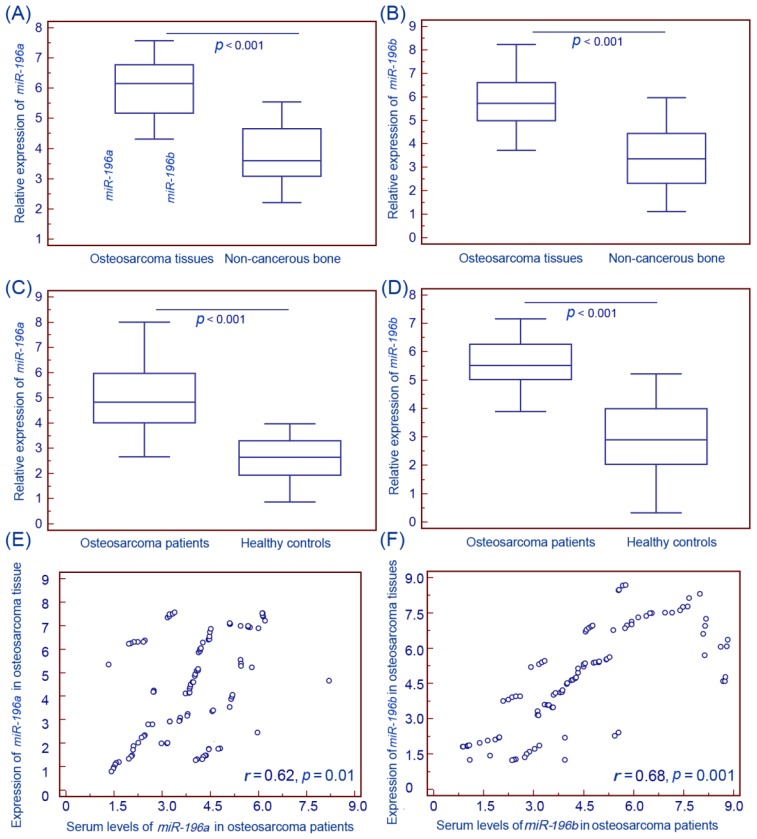
Expression levels of *miR-196a* and *miR-196b* in human osteosarcoma tissues and patients’ sera detected by qRT-PCR (Quantitative real-time reverse transcriptase-polymerase chain reaction) assay. The results showed that the expression levels of *miR-196a* (**A**) and *miR-196b* (**B**) in osteosarcoma tissues were both significantly higher than those in noncancerous bone tissues (both *p* < 0.001); Similarly, the serum levels of *miR-196a* (**C**) and *miR-196b* (**D**) were also markedly upregulated in patients with osteosarcomas compared with healthy controls (both *p* < 0.001); More interestingly, the expression levels of *miR-196a* and *miR-196b* in osteosarcoma tissues were both significantly correlated with those in patients’ sera (for *miR-196a*: Spearman’s correlation: *r* = 0.62, *p* = 0.01, (**E**); for *miR-196b*: Spearman’s correlation: *r* = 0.68, *p* = 0.001, (**F**)).

Expression levels of *miR-196a* and *miR-196b* in osteosarcoma and corresponding noncancerous bone biopsy samples, as well as in patients’ sera and healthy controls were detected by qRT-PCR and normalized to RNU6B (U6 snRNA). As the results, the expression levels of *miR-196a* and *miR-196b* in osteosarcoma tissues were both significantly higher than those in noncancerous bone tissues (both *p* < 0.001, Figure 1A,B). Similarly, the serum levels of the two miRNAs were also markedly upregulated in patients with osteosarcomas compared with healthy controls (both *p* < 0.001, Figure 1C,D). More interestingly, the expression levels of *miR-196a* and *miR-196b* in osteosarcoma tissues were both significantly correlated with those in patients’ sera (for *miR-196a*: Spearman’s correlation: *r* = 0.62, *p* = 0.01, Figure 1E; for *miR-196b*: Spearman’s correlation: *r* = 0.68, *p* = 0.001, Figure 1F). Hence, we investigated the clinical significance of *miR-196a* and *miR-196b* in osteosarcoma using their serum levels in the next sections.

### 2.2. Serum Levels of miR-196a and miR-196b Associate with Clinicopathological Features of Human Osteosarcoma

In order to evaluate the associations of serum levels of *miR-196a* and *miR-196b* with the clinicopathological features of osteosarcoma patients, the median values of *miR-196a* (4.86) and *miR-196b* (5.48) expression in sera of 100 osteosarcoma patients were used as the cutoff points to divide these patients into *miR-196a*-low (*n* = 43), *miR-196a*-high (*n* = 57), *miR-196b*-low (*n* = 48) and *miR-196b*-high (*n* = 52) expression groups. On this basis, 31 (31.00%) cases were both low expression of *miR-196a* and *miR-196b*, 40 (40.00%) cases were both high expression of *miR-196a* and *miR-196b*, 12 (12.00%) cases were *miR-196a*-low and *miR-196b*-high expression, and 20 (20.00%) cases were *miR-196a*-high and *miR-196b*-low expression.

As shown in Table 1, the upregulation of *miR-196a* and *miR-196b* both more frequently occurred in osteosarcoma patients with high tumor grade (*p* = 0.008 and 0.01, respectively), positive metastasis (*p* = 0.001 and 0.006, respectively) and recurrence (*p* = 0.001 and 0.006, respectively). Of note, the combined upregulation of *miR-196a* and *miR-196b* was also significantly associated with high tumor grade (*p* < 0.001), the presence of metastasis (*p* < 0.001) and recurrence (*p* < 0.001) of patients with osteosarcomas.

### 2.3. Serum Levels of miR-196a and miR-196b Predicts Prognosis in Patients with Osteosarcoma

**Figure 2.**
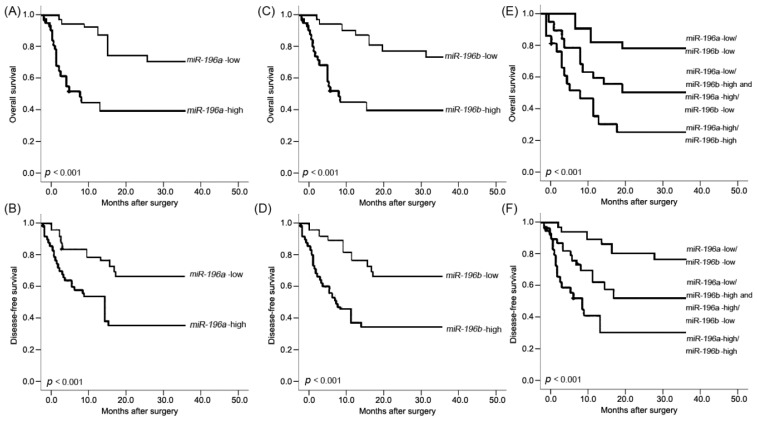
Kaplan-Meier survival curves for osteosarcoma patients according to *miR-196a* expression ((**A**) for overall survival; (**B**) for disease-free survival); *miR-196b* expression ((**C**) for overall survival; (**D**) for disease-free survival) and concomitant *miR-196a* and *miR-196b* expression (*miR-196a*/*miR-196b*, (**E**) for overall survival; and (**F**) for disease-free survival).

According to the results of Kaplan-Meier method and log-rank test, the patients with high *miR-196a* expression and high *miR-196b* expression both had shorter OS (both *p* < 0.001, Figure 2A,C) and DFS (both *p* < 0.001, Figure 2B,D) than those with high expressions. Of note, the OS and DFS of patients with combined *miR-196a* and *miR-196b* upregulation (*miR-196a*-high/*miR-196b*-high) were the shortest (both *p* < 0.001, Figure 2E,F) when compared with patients in other three groups (*miR-196a*-low/*miR-196b*-high, *miR-196a*-high/*miR-196b*-low, *miR-196a*-low/*miR-196b*-low). In addition, the OS and DFS benefits were also found in the patients with low tumor grade (*p* = 0.006 and 0.002, respectively), good response to pre-operative chemotherapy (both *p* = 0.02), and the absence of metastasis (both *p* < 0.001) and recurrence (both *p* < 0.001).

Cox proportional hazard model confirmed that *miR-196a* expression (for OS: RR 6.28, 95% CI, 1.62–13.39, *p* = 0.01; for DFS: RR 6.95, 95% CI, 1.63–14.61, *p* = 0.01), *miR-196b* expression (for OS: RR 6.33, 95% CI, 1.61–13.48, *p* = 0.01; for DFS: RR 6.98, 95% CI, 1.65–14.82, *p* = 0.01) and *miR-196a*/*miR-196b* expression (for OS: RR 9.89, 95% CI, 2.66–20.98, *p* = 0.001; for DFS: RR 10.09, 95% CI, 2.82–21.99, *p* = 0.001) were all independent prognostic factors of unfavorable survival in human osteosarcoma (Table 2).

### 2.4. Discussion

Multiple and complex genomic aberrations are implicated into osteosarcomagenesis. miRNAs have been proved as an efficient diagnostic and therapeutic tool in several human cancers. Depending on the target gene, miRNAs function as tumor suppressor genes or have an oncogenic role in cancer formation. Tumor-suppressive miRNAs often repress growth-promoting genes, and oncogenic miRNAs often target growth-stimulatory genes [17]. In the current study, we analyzed the expression of two miRNAs, miR-196a and miR-196b, in osteosarcoma tissue and serum samples for the association with clinicopathological and survival data from patients. We found that *miR-196a* and *miR-196b* expression were both upregulated in osteosarcoma tissues compared to the corresponding noncancerous bone tissues. Similarly, their serum levels were also significantly increased in osteosarcoma patients compared to levels in healthy controls. Moreover, we found that higher serum levels of *miR-196a* and *miR-196b* were associated with short OS and short DFS of osteosarcoma patients. Furthermore, the univariate and multivariate analyses showed that serum levels of *miR-196a* and *miR-196b*, and their combined serum levels were all independent predictors of OS and DFS of osteosarcoma patients.

Accumulating studies have demonstrated that *miR-196a* may be implicated in malignancy, but its role may differ among various cancer types. *miR-196a* is located in HOX gene clusters and potentially targets *HOXB8*, *HOXC8*, *HOXD8* and *HOXA7*, which have been proved to play important roles in oncogenesis [18]. The upregulation of *miR-196a* was observed in glioblastoma, esophageal cancer, non-small cell lung cancer, pancreatic cancer, gastric cancer, colorectal adenocarcinoma and endometrial cancer, but it is downregulated in breast cancer and melanoma [19–25]. Functionally, *miR-196a* has been found to promote cancer cell proliferation, detachment, migration, invasion of colorectal cancer and non-small cell lung cancer cell lines [20]; It can be used to correctly differentiate pancreatic cancer from benign pancreatic tissue, and high expression of *miR-196a* is found to predict poor survival [21]; Strong expression of *miR-196a* is also associated with a poor prognosis in patients with glioblastoma [22]; High *miR-196a* levels in gastric cancer patients’ sera may associate with the disease state and relapse potential [23]. In contrast, *miR-196a* can suppress invasion and metastasis of breast cancer cells [24], and also inhibit the invasive behavior of melanoma cells [25]. In the current study, our data showed the upregulation of *miR-196a* in both osteosarcoma tissues and patients’ sera, which was similar with the reports of Namløs *et al*. [16] on osteosarcoma cell lines. We also confirmed the significant association of *miR-196a* overexpression with aggressive tumor progression and poor prognosis of patients with this disease. These findings indicate that *miR-196a* may exert opposite effects in different tumors.

As another member of the *miR-196* cluster, the expression patterns and the roles of *miR-196b* have been demonstrated to be very controversial across various cancer types. *miR-196b* expression was increased in leukemia, flioblastoma, gastric cancer, pancreatic ductal adenocarcinoma and bronchial squamous cell carcinoma, but was reduced in cervical cancer [26–31]. Functionally, the expression of *miR-196b* increases cell proliferation and survival in leukemic cells [26]; The lack of promoter methylation in *miR-196b* may lead to its overexpression in gastric cancer cell lines and patient samples, which might provide a useful tumor marker [27]; The overexpression of *miR-196b* confers a poor prognosis via promoting cellular proliferation in glioblastoma patients [28]; In contrast, *miR-196b* downregulation may be significantly associated with worse disease-free survival for cervical cancer patients treated with combined chemo-radiation [31]. In the current study, the findings of *miR-196b* were very similar to *miR-196a*, suggesting that it might be also involved in malignant progression of osteosarcoma.

More interestingly, we here also analyzed the association of *miR-196a*/*miR-196b* conjoined expression with the prognosis of osteosarcoma. Significant difference of prognosis was found among four different statuses of *miR-196a*/*miR-196b* co-expression. The subjects with *miR-196a*-high/*miR-196b*-high had the worst OS and DFS, while the *miR-196a*-low/*miR-196b*-low had the best. Multivariate analysis revealed that the *miR-196a*/*miR-196b* co-expression profiles was an independent prognostic indicator for osteosarcoma. The advantages of *miR-196a*/*miR-196b* co-expression to individual *miR-196a* or *miR-196b* in predicting the outcome of osteosarcoma has been shown in our result.

## 3. Experimental Section

### 3.1. Ethics Statement

This study was approved by the Ethical Review Committee of the Second Hospital Affiliated to Xi’an Jiao Tong University School of Medicine, Xi’an, Shaanxi, China. All specimens were handled and made anonymous according to the ethical and legal standards and were obtained with patients’ written informed consent.

### 3.2. Patients and Tissue Samples

In total, we recruited 100 patients with osteosarcomas from Department of Orthopedic Surgery, the Second Hospital Affiliated to Xi’an Jiao Tong University School of Medicine, Xi’an, Shaanxi, China between February 2007 and May 2010. For tissue sample collection, 100 pairs of osteosarcoma tissues and corresponding noncancerous bone tissues were collected from the same patients. The tissues were removed from surgical specimens, immediately transported to the Pathology Laboratory, frozen and stored at −80 °C for RNA extraction. In addition, sera from all 100 patients with osteosarcomas before chemotherapy and 100 healthy volunteers matched according to sex and age were also collected. All tissue samples were reviewed by two pathologists (Dr. Li H. and Dr. Wang G.), and the clinicopathologic data such as age, sex, site, histologic type, tumor grade, surgical method, response to chemotherapy, and the status of metastasis and recurrence were retrospectively reviewed and summarized in Table 1.

Following the diagnosis, all 100 patients with osteosarcomas enrolled in this study were treated with the same neoadjuvant chemotherapy consisting of methotrexate (MTX), doxorubicin (ADM), cisplatin (CDP), and ifosfamide (IFO). All drugs were given intravenously. After that, all the patients underwent wide resection of tumor. Response to chemotherapy was classified as “poor” (<90% tumor necrosis) and “good” (>90% tumor necrosis) through histologic analysis of tumor specimens after surgery [32].

For the survival analysis, all 100 patients with osteosarcoma enrolled in this study received follow-up and were monitored with computed tomography (CT) performed every three months during the first three years after chemotherapy, every four months during years 4 and 5 and every six months thereafter. CT scans or magnetic resonance imaging (MRI) were performed to determine the development of local recurrence and distant metastasis. The median follow-up of this cohort was 30.8 months (range: 7.8–39.6 months). In this study, overall survival (OS) was defined as the time interval from the date of diagnosis at our center to the date of death or the last follow-up. Disease-free survival (DFS) was defined as the time interval from diagnosis at our center to progressive disease, death of any other cause than progression, or a second primary cancer.

### 3.3. RNA Extraction

Total RNA was isolated from fresh osteosarcoma tissues, corresponding noncancerous tissues, sera of osteosarcoma patients and healthy controls by using mirVana miRNA Isolation Kit (Ambion, Austin, TX, USA) according to the manufacture’s instruction. RNA concentration was determined using a NanoDrop ND-1000 spectrophotometer (NanoDrop Technologies, Wilmington, DE, USA), and RNA quality was measured using a denaturing 15% polyacrylamide gel.

### 3.4. QRT-PCR for miRNA

Reverse transcription (RT) reaction was performed using the TaqMan MicroRNA Reverse Transcription Kit (Applied Biosystems, Foster City, CA, USA) according to the manufacturer’s instructions and 10 ng total RNA was utilized in the RT reactions. Following the detection with Applied Biosystems prism 7500 Real-Time PCR System (Applied Biosystems), qRT-PCR was performed using specific TaqMan MicroRNA analysis (Applied Biosystems). RNU6 (Applied Biosystems) was used as the endogenous control for the expression of *miR-196a* and *miR-196b*. Real-time PCR reactions for miRNAs were performed in triplicate in 20 μL volumes. The sequences of the primers were as follows: *miR-196a* forward, 5′-CGTCAGAAGGAATGATGCACAG-3′; reverse, 5′-ACCTGCGTAGGTAGTTTCATGT-3′; *miR-196b* forward, 5′-TAGGTACCACTTTAT CCCGTTCACCA-3′; reverse, 5′-ATCTCGAGGCAGGGAGAGAGGAATAA-3′; *U6* forward 5′-CTC GCTTCGGCAGCACA-3′ and reverse 5′-AACGCTTCACGAATTTGCGT-3′. Quantitative miRNA expression data were acquired and analyzed using an Applied Biosystems 7500 real-time PCR system (Applied Biosystems). The qRT-PCR assays for a particular gene were undertaken at the same time for all samples under identical conditions, in triplicate. The miRNA relative expression was calculated using a 2^−ΔΔ^*^C^*^t^ method [33].

### 3.5. Statistical Analysis

Statistical analysis was performed by using the software of SPSS version 13.0 for Windows (SPSS Inc., Chicago, IL, USA). Continuous variables were expressed as mean ± S.D. A paired sample *t*-test was used to compare differences in miRNA expression between osteosarcoma tissues and non-cancerous bone tissues. The correlation of *miR-196a* or *miR-196b* expression between osteosarcoma tissues and serum tissues was determined by Spearman Correlation analysis. The statistical significance of the correlation of *miR-196a* and *miR-196b* expression with various clinicopathological parameters was evaluated by Fisher’s exact test or x2 test. The Kaplan-Meier test was used to determine the probability of survival and data were analyzed by the log-rank test. Differences were considered statistically significant when *p* was less than 0.05.

## 4. Conclusions

Our present data indicate the involvement of *miR-196a* and *miR-196b* upregulation in the pathogenesis of osteosarcoma. More importantly, the altered levels of circulating *miR-196a* and *miR-196b* might have great potential to serve as novel and non-invasive prognostic factors for this malignancy. Further investigation extended in much more cases is in need to evaluate the potential application value of *miR-196a*, *miR-196b*, and *miR-196a*/*miR-196b* conjoined expression, as dependent or independent prognosis factor/s of osteosarcoma, in a clinical setting.

## Figures and Tables

**Table 1.** Association of serum miR-196a and miR-196b levels with clinicopathological features of osteosarcoma.

Clinicopathological Features	No. of Cases	*miR-196a*-High (*n*, %)	*p*	*miR-196b*-High (*n*, %)	*p*	*miR-196a*-High/*miR-196b*-High (*n*, %)	*p*
*Age*							

<18	32 (32.00)	20 (62.51)	NS	14 (43.75)	NS	12 (37.50)	NS
≥18	68 (68.00)	37 (54.41)		38 (55.88)		28 (41.18)	

*Sex*							

Male	70 (70.00)	40 (57.14)	NS	36 (51.73)	NS	28 (40.00)	NS
Female	30 (30.00)	17 (56.67)		16 (53.33)		12 (40.00)	

*Tumor Site*							

Femur	58 (58.00)	35 (60.34)	NS	30 (51.72)	NS	22 (37.93)	NS
Tibia	20 (20.00)	10 (50.00)		11 (55.00)		9 (45.00)	
Humeral bone	15 (15.00)	8 (53.33)		8 (53.33)		6 (40.00)	
Other	7 (7.00)	4 (57.14)		3 (42.86)		3 (42.86)	

*Histologic Type*							

Osteoblastic	52 (52.00)	30 (57.69)	NS	28 (53.85)	NS	20 (38.46)	NS
Chondroblastic	18 (18.00)	10 (55.56)		9 (50.00)		8 (44.44)	
Fibroblastic	20 (20.00)	11 (55.00)		10 (50.00)		8 (40.00)	
Telangiectatic	10 (10.00)	6 (60.00)		5 (50.00)		4 (40.00)	

*Tumor Grade*							

Low	38 (38.00)	15 (39.47)	0.008	12 (31.58)	0.01	8 (23.53)	<0.001
High	62 (62.00)	42 (67.74)		40 (64.52)		32 (51.61)	

*Metastasis*							

Absent	60 (60.00)	30 (50.00)	0.001	26 (43.33)	0.006	12 (20.00)	<0.001
Present	40 (40.00)	27 (67.50)		26 (65.00)		28 (70.00)	

*Recurrence*							

Absent	65 (65.00)	27 (41.54)	0.001	26 (40.00)	0.006	14 (21.54)	<0.001
Present	35 (35.00)	30 (85.71)		26 (74.29)		26 (74.29)	

*Response to Pre-Operative Chemotherapy*							

Good	60 (60.00)	31 (51.67)	NS	32 (53.33)	NS	22 (36.67)	NS
Poor	40 (40.00)	26 (65.00)		20 (50.00)		18 (45.00)	

**Table 2.** Multivariate survival analysis of overall survival (OS) and disease-free survival (DFS) in 100 patients with osteosarcoma.

Variables	OS			DFS		
	
	RR	95% CI	*p*	RR	95% CI	*p*
Tumor grade	6.86	1.69–15.02	0.01	7.37	1.72–16.31	0.008
Response to pre-operative chemotherapy	3.58	0.80–7.21	0.04	4.02	1.01–8.38	0.03
Metastasis status	3.19	0.76–6.82	NS	3.56	0.80–7.19	0.04
Recurrence status	3.48	0.79–7.03	NS	4.00	1.00–8.06	0.03
Serum miR-196a level	6.28	1.62–13.39	0.01	6.95	1.63–14.61	0.01
Serum miR-196b level	6.33	1.61–13.48	0.01	6.98	1.65–14.82	0.01
miR-196a/miR-196b expression	9.89	2.66–20.98	0.001	10.09	2.82–21.99	0.001
